# Framing the detection of incipient tuberculosis infection: A qualitative study of political prioritisation

**DOI:** 10.1111/tmi.13734

**Published:** 2022-02-22

**Authors:** Rosemary James, Grant Theron, Frank Cobelens, Nora Engel

**Affiliations:** ^1^ Department of Health, Ethics & Society Maastricht University Maastricht The Netherlands; ^2^ Clinical Mycobacteriology and Epidemiology Group Stellenbosch University Stellenbosch South Africa; ^3^ Department of Global Health and Amsterdam Institute for Global Health and Development Amsterdam University Medical Centers Amsterdam The Netherlands

**Keywords:** global health, health policy, health priorities, latent tuberculosis, qualitative methods, tuberculosis

## Abstract

**Objective:**

Incipient Tuberculosis (ITB) refers to *Mycobacterium tuberculosis* infection that is likely to progress to active disease in the absence of treatment, but without clinical signs, symptoms, radiographic or microbiological evidence of disease. Biomarker‐based tests to diagnose incipient TB hold promise for better prediction and, through TB preventive therapy, prevention of disease. This study explored current and future framing and prioritisation of ITB.

**Methods:**

Twenty‐two interviews across eight countries were conducted. A modified Shiffman & Smith Framework, containing four categories—Ideas, Issue Characteristics, Actor Power, and Political Contexts—was used to analyse the current landscape and potential for prioritisation of diagnosis and treatment of ITB.

**Results:**

Latent TB policy implementation has been slow due to technical, logistical and financial challenges, and because it has been framed in a manner non‐conducive to gaining political priority. Framing ITB testing as ‘early detection’ rather than ‘prediction’, and its management as ‘treatment’ rather than ‘preventive therapy’, may help raise its importance in policies, and its acceptance among actors.

**Conclusion:**

Consensus surrounding the framing of ITB will be crucial for the successful adoption of ITB diagnostics and treatment. When designing ITB tools and policies, it will be important to address challenges that pertain to latent TB policies.

## INTRODUCTION

Nearly one‐quarter of the world's population is thought to be infected with *Mycobacterium tuberculosis*, yet only an estimated 10% of those infected develop tuberculosis (TB) during their lifetime [1]. To prevent the occurrence of TB, *M*. *tuberculosis* infection can be treated with preventive therapy (TPT). Drug combinations such as isoniazid‐rifampicin or isoniazid‐rifapentine for 3 months or less have demonstrated promising efficacy and cost‐effectiveness [2, 3, 4]. One of the greatest challenges pertaining to TB infection is to accurately predict who will progress to active disease and benefit from TPT. There is an ongoing paradigm shift in TB control resulting from discoveries around the spectrum of TB infection, that is, the different immunological and clinical stages that a person goes through and their timing. This spectrum is thought to have at least four mutually exclusive stages; latent TB infection (LTBI), incipient TB (ITB), subclinical TB, and active disease. Subclinical TB is an asymptomatic disease state of which the transmissibility is not well understood [5], whereas LTBI refers to a state of persistent containment of *M*. *tuberculosis* replication without evidence of TB‐related pathology [6] ITB is a transitional state between LTBI and disease. A relatively new concept, it refers to *M*. *tuberculosis* infection that is likely to progress to active disease in the absence of treatment but without clinical signs, symptoms, radiographic or microbiological evidence of disease [7]. It is thought that 80% or more of progression from LTBI to active disease is within 2 years, but who progresses, how, and why is not yet fully understood [8].

The ability to detect and treat ITB would be beneficial‐ to scale up contact tracing, as it requires fewer people to be offered TPT, and to prevent further transmission, disease and suffering. Although several biomarkers have been identified that can predict progression to active TB, primarily reflecting inflammatory responses, there are currently no commercially available tools to diagnose ITB [7, 9, 10, 11, 12]. The profile of those who would be targeted for screening, and what treatment regimens and follow‐up would be most effective is yet to be determined, as is how people might respond to being diagnosed at this new stage of infection. TPT policies depend on resources and perceived impact on the TB incidence at the country level, and the presence of risk factors for developing TB disease at the individual level. Whether ITB policies should be framed similarly or differently from existing TPT policies is currently in discussion.

ITB is an emerging topic that has yet to receive widespread political attention, while the related issue of LTBI has suffered from challenges in prioritisation. To avoid similar challenges, it is important to understand how ITB is framed by those involved in research, advocacy, and policymaking, and with what implications for both proposed solutions, for example, screening programmes, diagnostics, treatment solutions, and other forms of support, and their likely prioritisation in the face of other pressing concerns.

### Where are we now?

For the first time in history, WHO is advocating for global investment in the early detection of TB in high‐risk groups globally [13, 14]. Many countries already include LTBI screening and TPT in their national TB strategies and are working to expand them per the latest WHO recommendations [15, 16]. Breakthroughs in biomarkers that may predict progression to disease could further advance these policies. In 2015, the Stop TB ‐ Foundation for Innovative New Diagnostics (FIND) – Stop TB New Diagnostics Working Group (NDWG) convened a workshop to discuss the development of tests to predict the progression of LTBI to active disease. In 2016, they established a ‘Task Force on LTBI and test of progression’, the same year that a prospective cohort study identified a 16‐transcript blood signature capable of predicting the progression TB [11]. In 2017, WHO published a Target Product Profile (TPP) for a test to predict the progression of TB infection [17, 18]. Most recently, WHO held a technical consultation on LTBI management in Montréal in 2019, where the potential for ITB diagnostics was touched upon [19]. Until recently, LTBI has been neglected in TB policies due to the prioritisation of active TB, and limited diagnostic and treatment options [20]. With recently updated WHO LTBI guidelines [14] and shorter drug regimens, it is expected that many countries will begin to introduce LTBI screening for multiple risk groups. However, many challenges surround LTBI diagnostics and treatment.

### Existing LTBI challenges

Latent TB infection diagnostics, the tuberculin skin test (TST) or interferon‐gamma release assays (IGRAs), have limited value in predicting progression to active TB, meaning that mass screening for LTBI is neither practical nor cost‐effective [21, 22]. A review of 98 national TB policies revealed that although most countries do acknowledge LTBI, most lack budget and indicators for its monitoring and evaluation, making it difficult to improve its programmatic management [16]. A survey of high‐burden countries found that few have LTBI guidelines that are fully implemented [15]. Upstream barriers, such as lack of national strategy, political commitment, and health worker regulation, make implementation difficult [23]. In high‐burden settings, the accessibility and cost of IGRAs and new treatments, a shortage of purified protein derivative (PPD) for TSTs, lack of regulatory approval for new drugs, the risk of re‐infection, and a focus on active TB in national programmes are also current challenges [4, 15, 24]. System failures in following‐up with TB contacts [25], hesitancy of physicians to prescribe preventive therapy [24, 26, 27], and inadequate workforce allocation of time and training [28, 29] are also recognised. There is also inadequate uptake of TPT particularly in people living with HIV, thought to be due to lack of counselling and support [15, 23, 30, 31]. Furthermore, drug adherence has been historically poor [32, 33, 34]; however, the recent introduction of shorter regimens has shown improved rates of treatment completion among people with LTBI [34, 35]. Follow‐up is challenging, particularly for migrants and rural populations, due to poor access and return appointments that are required for TSTs [24, 36]. Additionally, there are ethical dimensions, for example, whether it is fair to have low‐risk people go through the process of screening for LTBI, as tests have low predictive values that could lead to unwarranted stress and prejudice, and unnecessary treatment [37, 38, 39].

### Objective

The purpose of this study was to explore how the concept of ITB, including its definition, and proposed screening, diagnosis, management and follow‐up, is being framed by TB researchers, policymakers and healthcare providers, to inform future policies, and to evaluate its potential for political prioritisation in comparison to that of LTBI. We explored future opportunities and challenges that ITB tools may encounter once brought to market, by applying a policy analysis framework to interviews conducted with those knowledgeable of ITB.

## METHODS

We used qualitative methods to study the framing of ITB in policy and research. Semi‐structured interviews were conducted with a global variety of ITB experts (Table 1) such as researchers, policymakers, and healthcare providers from both high‐ and low‐burden TB countries. Purposeful sampling was used to ensure diversity in gender, profession and geographical location. Respondents were recruited by using the study team's professional networks, and through contacting authors of recent ITB publications and studies. Using a snowball approach, earlier respondents were asked to suggest further respondents.

**TABLE 1:** Selection criteria for interview respondents

Type	Eligibility criteria
Policymakers and policy advisors	Has an interest in ITB Active (or has previously worked) on creating or advising on policies surrounding TB, preferably asymptomatic phases ≥18 years of age Fluent in English or French
Researchers	Has an interest in ITB Has conducted (qualitative or quantitative) research surrounding TB, preferably asymptomatic phases ≥18 years of age Fluent in English or French
Healthcare providers	Experience in working with TB patients or TB contact‐tracing ≥18 years of age Fluent in English or French

### Data collection

Due to the COVID‐19 pandemic, interviews took place via Zoom lasting on average between 40 and 60 min. All interviews were carried out by the primary investigator. An exploratory interview guide of open‐ended questions based on the study framework, background literature review, and expert opinion was used. The guide was kept broad to avoid investigator bias and was adapted for each respondent based on their area of expertise. The interview questions revolved around four main domains: challenges pertaining to LTBI and TPT, how the spectrum of TB is understood, the prioritisation of asymptomatic TB in policies, and considerations for future ITB policies. All interviews were audio‐recorded using a portable offline device and were transcribed verbatim.

### Data analysis

The Shiffman & Smith Framework [40] (Table 2) was applied to explore the current and future political prioritisation of ITB. The framework considers both internal and external framing as well as the political economy as factors for shaping the policy and subsequent prioritisation of the portrayed problem [41]. It is considered the most comprehensive health policy framework [42], as both internal and external powers are analysed, in comparison to more focused frameworks that do not always consider the environment outside of the scope. We slightly modified the framework, similar to an earlier adaptation [43], by altering the order and factors of the categories, to fit the sequence of the questions in the interview guide, and to flow from an internal level to a more external environmental level [44].

**TABLE 2:** Study Framework (adapted from Shiffman & Smith [40])

Category	Factors shaping political priority	Description
Ideas (the ways in which actors understand and portray the issue)	Internal framing	The degree to which the policy community agrees on the definition of, causes of, and solutions to the problem
	External framing	Public portrayals of the issue in ways that resonate with external audiences, especially the political leaders who control resources
Issue characteristics (features of the problem)	Severity	The size of the burden relative to other problems, as indicated by objective measures such as mortality levels
	Credible indicators	Clear measures that demonstrate the severity of the problem and that can be used to monitor progress
	Effective interventions	The extent to which proposed means of addressing the problem is clearly explained, cost‐effective, backed by scientific evidence, simple to implement and inexpensive
Actor power (the strength of the individuals and organisations concerned with the issue)	Guiding institutions	The effectiveness of organisations or coordinating mechanisms with a mandate to lead the initiative
	Leadership	The presence of individuals capable of uniting the policy community and acknowledged as a particularly strong leaders for the cause
	Civil society mobilisation	The extent to which grassroots organisations have mobilised to press international and national political authorities to address the issue at the global level
Political contexts (the environments in which actors operate)	Global governance structure	The degree to which norms and institutions operating in a sector provide a platform for effective collective action
	Policy community cohesion	The degree of coalescence among the network of individuals and organisations centrally involved with the issue at the global level
	Policy windows	Political moments when global conditions align favourably for an issue, presenting opportunities for advocates to influence decision‐makers

The transcripts were read in entirety by the first author and uploaded to Atlas.ti 8.0 software for inductive and deductive coding. Using thematic analysis, a code list was developed based on the study framework. The four domains did not fit well into the framework—and were thus coded separately. The arising themes of these domains were refined and sorted into a scheme of 46 code groups and 30 subcodes (Table S2). The final themes were analysed using Atlas.ti maps, with sub‐themes and interview quotations attached.

### Ethical considerations

Ethical approval was obtained from Maastricht University's Faculty of Global Health ethical review board for the Global Health Masters programme. Prior to the interviews, respondents were required to read a participant information sheet and sign a consent form, including optional consent to be audio recorded.

## RESULTS

A total of 22 semi‐structured interviews were conducted between May and August 2020. Respondents had 2–35 years' experience with TB and resided in eight countries spanning Asia, Africa, Europe and North America (Table S1).

Overall, results indicated that the TB community has yet to fully understand and define the full spectrum of TB infection. Current challenges surrounding TPT implementation, including cost and accessibility of diagnostics and treatment, lack of designated human resources and low treatment adherence rates should be considered for the future development of ITB policies and implementation. Framing ITB as early detection and treatment rather than as prevention could be conducive to gaining political prioritisation. Furthermore, for ITB diagnostics to be successful, policies should be adaptable to context, considering local prevalence, cultural norms and resources. Table 3 outlines the overarching policy challenges and opportunities identified from the interviews.

**TABLE 3:** Challenges and prospects for the prioritisation of incipient TB in policies

Category	Challenges	Prospects	Illustrative quote
Ideas	No clear consensus on definitions of the spectrum Public portrayal is largely negative or absent Poor framing of LTBI and ITB	Framing ITB screening and diagnosis as ‘early detection’ rather than ‘prevention’ could increase its prioritisation Respondents agreed tests that can predict the progression of TB are considered crucial for TB elimination Research is ongoing	*‘When I work in latent TB, what I always try to say is… ‘it's not about preventing TB, it's about also curing an infection’. So many times, the message of cure is much more powerful at the level of society. Even at programme level, (…) the policymakers (…) will decide, depending on the money availability, for strong interventions that call for treatment and cure and not so much prevention. So, I personally don't use very much ‘TB prevention’ for example. So I talk about treatment for infection and treatment for disease'*. [Global TB Policy Advisor, Interview#13]
Issue characteristics	Indicators of the burden and interventions to address ITB do not yet exist Lack of funding Many research gaps Crossover to existing LTBI challenges	Recent study results are showing promising biomarkers which could fit the TPP Cost‐effectiveness of treating ITB Framing ITB as early detection rather than prevention	*‘I think it's about convincing people of the value of testing. In that, this is a cost‐effective intervention. And that now we have a higher predictive value that people are going to get sick and that it's better to treat them before they do!’* [TB Researcher, Interview #3]
Actor power	Minimal civil society engagement No individual or country leaders	FIND‐Stop TB NDWG platform for internal discussions Increasing civil society engagement regarding LTBI preventive therapy Civil society and funder involvement in ITB research, development, and rollout of tools Community engagement	*‘Mass screening only works if you have strong community mobilisation led by people from the communities, designed by people from the community, and supported. So, for me, something like [an ITB test], is the question of whether… it's not the test that's going to succeed or fail. It's your process surrounding the test or the screening that's going to succeed or fail. For me, the issue is the design and the inclusion processes of the design, rather than the test itself’*. [TB Anthropologist, Interview#5]
Political contexts	No global governance or coordination mechanism to date Global issues such as COVID‐19 could divert focus away from TB	The updated 2020 WHO Guidelines on LTBI and 2018 UN high‐level meeting on TB have created a policy window for action	*‘The field is moving forward and you know, technology sometimes moves really rapidly. I mean, look at what COVID−19 is showing us. When there's urgency, things can happen. And so, I mean, certainly the technology I mean, nothing is impossible and the technology is all there. It's all possible. It's just about whether people can actually raise the capital and kind of put together everything to achieve it in a faster frame of time’*. [TB Researcher, Interview #8]

### Ideas

Respondents agreed that a test to predict progression of TB infection is widely desired, as such an advancement would be transformational to scaling‐up TPT and meeting the WHO's End‐TB Strategy's targets [13], by being able to determine more accurately who requires treatment. Although they had a basic awareness and understanding of the spectrum of TB, most respondents acknowledged they were not familiar with ITB and lacked a clear understanding of the concept. Respondents expressed confusion surrounding ITB regarding the anatomical location where *M*. *tuberculosis* resides, whether an IGRA response would be positive, whether chest radiography has any role if biomarkers have been validated, and whether individuals could progress or regress along the spectrum of disease.

Differences in opinions existed between researchers, clinicians and policymakers interviewed. In particular, those who were not involved in test development had assumptions that accurate tests would soon be available and were more concerned regarding their future implementation. Scientists involved with ITB research were more hesitant to assume a test of progression would meet the TPP criteria anytime soon and may require different biomarkers for different groups. Some respondents viewed future ITB diagnostics under the realm of ‘personalised medicine,’ where different host responses based on local epidemiology and genetics equate to different biomarkers, therefore requiring different tests for different settings and individuals. Respondents stressed the importance of defining the appropriate application of ITB tests based on the setting, health system structure, and prevalence of TB infection. For example, people living with HIV in high‐burden settings would be eligible for TPT without the need for testing [14]. A common idea was that ITB diagnostics should be usable at point‐of‐care and integrated into primary healthcare structures and systems, alongside other disease preventive and health promotive services, with minimal staff training. However, respondents recognised challenges to this integration, surrounding the ownership of the diagnostic tools, workforce allocation and the provision of counselling. Convincing asymptomatic people to start treatment is a challenge for LTBI [45, 46]—it could be for ITB as well unless risk communication is integrated into the model of care.

Most respondents agreed that to date, awareness of the spectrum of TB among the public is largely lacking. Many TB infections are asymptomatic, thus invisible to policymakers, healthcare providers, and the general public. Many patients are unaware of the concept of LTBI, why treatment and contact tracing are important, and what factors are involved with the risk of progression, for example [32]. Respondents with experience in advising on policies relating to TB control signalled that it is important to consider framing ITB diagnosis as ‘early detection’, and its subsequent management as ‘treatment’, ‘preventive treatment’ or ‘cure’, rather than framing it as ‘prevention’. They believed that policymakers and patients alike will be more likely to invest if they understand the disease as imminent and treatment as necessary, rather than as something that might potentially need preventative action in a percentage of the population. They agreed that the terminology for LTBI, such as the widely recognised *TPT* or isoniazid *preventive* treatment will have to be adjusted for future ITB management. A policy advisor highlighted how framing LTBI treatment as a ‘cure’, rather than as commonly termed, ‘preventive therapy’, helps to convince policymakers that it is worth investing in.

Respondents agreed that the media and civil society have an important role to play in the way ITB is understood and perceived by the public. Because ITB is asymptomatic, respondents were skeptical that the public would take interest in such a condition if they are unable to see the direct effects of its burden and acknowledged their vital role in advocating for equitable access to new diagnostics and treatments and spreading information about the symptoms of TB to promote early detection and decrease stigma.

### Issue characteristics

The burden of ITB is unknown, as validated biomarkers to detect ITB are not yet used in practice. For this reason, credible indicators and effective interventions do not yet exist. Hence, this aspect of the framework was largely absent. However, all respondents recognised the high global prevalence of LTBI, with many alluding to modelling projections that an estimated one‐quarter to one‐third of the global population is infected with *M*. *tuberculosis* [1, 44]. As ITB is asymptomatic, it was not considered as clinically severe in relation to other health issues. Multiple respondents considered drug‐resistant TB a larger, more pressing issue than that of detecting progressors, for example. Many predicted that it will be at least 5–10 years before an effective diagnostic to predict the progression to active disease becomes available, as did a recent WHO meeting consensus [19]. Cost‐effectiveness and accuracy of diagnostics were common qualities that respondents expressed to be important when addressing ITB—to gain political priority but also to ensure implementation in low‐resource settings is feasible. In terms of effective treatments, respondents agreed that recent advances in shorter regimens offer hope for effective management of ITB in future.

### Actor power

Leadership was found to be important at all levels—from WHO and other multilateral organisations to community‐level healthcare providers. The NDWG, comprised of academics and supported by the WHO TB programme, was perceived by respondents as influential in terms of shaping the global ITB research agenda and the WHO TPPs. Furthermore, some respondents remarked it will be important to apply lessons learnt from Unitaid and the Global Fund to fight AIDS, Tuberculosis and Malaria, which were largely responsible for reducing the price of GeneXpert and TB drugs, to ensure equitable access to ITB diagnostics. For example, Unitaid, in conjunction with the Clinton Health Access Initiative, recently succeeded in scaling‐up short‐course preventive treatment, dramatically cutting the price of rifapentine by partnering with an Indian pharmaceutical company [47, 48]. The role of the private sector was acknowledged by some respondents, particularly in terms of bringing new diagnostics to market. Downstream, local actors (politicians, health workers, community health leaders) were portrayed as vital for decision making and policy implementation, once tools are available. Awareness and education are important to improve physicians' knowledge about LTBI, such as the updated risk categories for which the WHO recommends testing, algorithms for testing, as well as their confidence in treatment. Most respondents believed that many physicians are not comfortable or equipped to meet the updated WHO LTBI guidance, due to uncertainties surrounding diagnostic predictive value, and treatment pitfalls, such as drug resistance, drug–drug interactions, and side effects [14]. In terms of the roll‐out, community leadership from key health workers and societal figures was deemed crucial. Mobilisation of the community in which the test would be targeting was an important consideration by respondents.

### Political Contexts

In the current landscape, the only policy community cohesion around ITB stems from the NDWG. However, the ambitious elimination targets set by the WHO's End‐TB Strategy [13], the UN General Assembly High‐Level Meeting on TB in 2018 [49] as well as the 2020 update of the WHO's LTBI guidance [14], were noted by respondents to be largely framing the shift in health policy movements toward effective early TB detection and prevention. An increase in global migration was an additional factor identified—provoking interest in TB screening, particularly in low‐incidence settings. WHO now recommends that migrants arriving from high‐burden countries are screened for LTBI [14]. Leadership from TB policy advisors in pushing ITB research was also said to be important, as they often have leverage and influence over national TB programmes. In terms of meeting the WHO TPP criteria for a test that can predict disease progression, most respondents viewed WHO guidance as imperative but stressed that it is ultimately research institutions, the private sector and policymakers who hold leverage for action. Overall, the policy windows for ITB are colliding, with global goals, migration and the COVD‐19 pandemic providing a platform, interest and motivation for advancing the field. The upcoming second UN high‐level meeting on TB, taking place in 2023 [50], could also be an opportunity to continue the paradigm shift of investing in the prevention of TB disease and put ITB on the global political agenda.

## DISCUSSION

Our findings reveal important gaps in research, policy, and practical guidance that should be addressed before accurate, cost‐effective, and acceptable ITB diagnostic and treatment protocols can be rolled out. Framing, that is, how something is portrayed or conceptualised, of a newly defined global health issue can dictate how it is prioritised [51, 52, 53]. How we recognise, define, and categorise disease states heavily influences the subsequent proposed management and policymaking surrounding the issue [54].

### Concepts of early TB

Only recently there is a wide understanding among experts that a broad spectrum of *M*. *tuberculosis* infection exists [55] Prevention and early detection of TB is a shifting paradigm as the spectrum becomes better understood [56]. Incipient is an adjective to describe something that is beginning to develop and originates from Late 16th century Latin, from the verb incipere, meaning ‘into, towards’ [57]. It was not until 2011 that the term was proposed to distinguish incipient infection from that of subclinical disease [58]. As of now, most TB infections are classified as active or latent, with little in‐between. Most definitions in literature do not specifically mention that it is possible individuals with ITB will either not progress, or even regress, along the spectrum of infection. However, the natural history of the spectrum of disease is becoming better understood, and a new state of minimal (early, non‐infectious) disease is also being considered [59]. This is an important caveat, as there could be a subset of individuals who will be prescribed treatment unnecessarily, as with currently available LTBI diagnostics. However, it is expected that the proportion of people treated unnecessarily will be lower than with LTBI diagnostics as ITB tests will pick up the infection further down the spectrum [7, 10, 18, 56, 58, 60, 61, 62].

This study found that terminology is important, not only for defining the spectrum among the scientific community to enhance research and development (R&D) efforts but also for determining how ITB is portrayed to the public and policymakers. External framing is important at a policy level, for priority setting, and securing access to medicines and diagnostics, while at a user level, it is important for encouraging health‐seeking behaviour and adherence to treatment. Civil society, including the media, also plays an important role here in generating support and demand toward eliminating TB, and should be conscious of the way they frame early infection. Framing ITB diagnosis as ‘early detection’, and its subsequent management as ‘treatment’, rather than ‘prevention’ may be vital in securing support. Although the terminology is important, especially once ITB tools become publicly available, a consensus on terms will require further understanding of the spectrum of TB. Further, the implications of potentially reframing treatment of ITB as ‘cure’ rather than ‘prevention’ merits further field research. This, however, should not delay the prioritisation of research and development for ITB tools. At present, the NDWG is leading the ITB research and development dialogue. The importance of allowing civil society to engage in the development of policy guidance was stressed by many respondents.

### Factors noted to be important in generating prioritisation

Most respondents emphasised the importance of learning from LTBI programme successes and challenges to maximise the future success of ITB diagnostic roll‐out. To address high‐level barriers, it will be important to increase political commitment and investment to attain country‐specific targets, public‐private collaboration, and continued investment in R&D for new and improved diagnostics and treatment [23, 63]. The colliding policy windows for ITB create a global policy arena that presents opportunities and a platform for stakeholders to justify their work and influence future policies. Respondents believed the implementation of ITB diagnostics needs to be context specific. Policies will have to be adaptable to each setting, largely based on the local TB prevalence, social norms and beliefs, and health system resources. In the meantime, our findings demonstrate that discussions surrounding the future implementation of ITB policies should begin imminently to secure access to future tools and treatments and plan for their health system and community integration. Recent civil society successes in improving access to LTBI preventive therapy, such as Unitaid and activist groups reducing barriers to drug manufacturing and pricing [47, 48], should be learnt from and applied to the research, development and rollout of ITB tools.

The framework was useful in identifying certain research gaps, based on aspects of the categories that were left unclear from the interview data, such as the severity of the issue in comparison to other issues, and effective interventions. However, there are several limitations to this study. The framework we used focused on the political prioritisation of ITB, as this has previously not been investigated. More exploratory studies are warranted, to uncover further challenges and perspectives surrounding the disease state. Furthermore, it was challenging to apply the framework prospectively—anticipating future as well as current needs, differentiating political priority from healthcare priority, and distinguishing between the four actor categories. There may be selection bias of the respondents, as purposeful rather than random sampling was used. Only people who identify as experts in latent or incipient TB were approached to be interviewed. Few respondents were based in high incidence, low‐income settings. Furthermore, allied health professionals, people with TB, and the public were not included in this study. Finally, due to the online virtual format and limited time of the interviews, there is a chance that important details were missed, or were subject to observer bias [64, 65].

It will be important to avoid challenges that pertain to LTBI policy implementation, by learning from programmatic experiences and conducting field research with diverse populations, including healthcare providers and TB survivors, in a variety of setting, to better understand their experiences and potential concerns. It is also critical to consider how ITB will be framed to increase the likelihood that future policies and programmes are implemented and received successfully. We caution to be mindful of the ramifications of labelling people with a disease. Social scientists should be involved in future ITB trials to uncover challenges and risks that may arise from diagnosing people with a new stage of infection—as a diagnosis can ‘vindicate and blame, can legitimise or stigmatise, can facilitate access to resources just as it can restrict opportunities. A diagnosis can be welcomed or eschewed’ [66]. While detection and treatment of ITB offers great prospects for reducing TB disease, technological advances and more insights from qualitative research will be essential for success.

## Supporting information

Table S1‐S2
