# Supplementary material for: Framing the detection of incipient tuberculosis infection: A qualitative study of political prioritisation
Source: Trop Med Int Health. 2022 Feb 22;27(4):445–53. doi: 10.1111/tmi.13734 (PMC9306665; doi:10.1111/tmi.13734)
Supplement: Supplementary file 1 — Table S1‐S2 [file TMI-27-445-s001.docx]

**Table S1.** Respondent demographics

| **Interview number** | **Primary job type** | **Gender** | **Primary country of employment** |
| --- | --- | --- | --- |
| 1 | Clinical TB Researcher | Male | Uganda |
| 2 | Clinical TB Researcher | Male | India |
| 3 | Clinical TB Researcher | Female | India |
| 4 | Clinical TB Researcher | Female | Canada |
| 5 | TB Anthropologist | Female | South Africa |
| 6 | Clinical TB Researcher | Female | Canada |
| 7 | Clinical TB Researcher | Male | UK |
| 8 | Clinical TB Researcher | Male | South Africa |
| 9 | Global TB Policy Advisor | Male | UK |
| 10 | Clinical TB Researcher | Female | Canada |
| 11 | Clinical TB Researcher | Male | UK |
| 12 | Global TB Policy Advisor | Female | Switzerland |
| 13 | Global TB Policy Advisor | Female | Switzerland |
| 14 | TB Epidemiologist | Male | Greece |
| 15 | TB Policymaker | Male | Canada |
| 16 | Global TB Policymaker | Male | Switzerland |
| 17 | Global TB Policy Advisor | Male | Switzerland |
| 18 | Global TB Policymaker | Male | Switzerland |
| 19 | TB Policymaker | Male | South Africa |
| 20 | Global TB Policy Advisor | Female | Switzerland |
| 21 | Public Health Nurse | Female | Canada |
| 22 | Private Sector (R&D) | Female + Male | France |

**Table S2.** Coding scheme

| **Theme** | **Code groups and subcodes** |
| --- | --- |
| **Latent Tuberculosis** | |
| Current challenges | Inability to predict progression  Under-prioritization  Treatment adherence  Contact tracing  Follow-up  Lack of resources   - Health workforce - Diagnostics - Treatment   Stigma  Counselling |
| Spectrum of TB | Understanding of the spectrum  Historical perspective (natural history)  Timeline  Thresholds/cut-offs  Importance of defining the spectrum |
| **Framework areas** | |
| Internal Framing | Definition of subclinical disease  Definition of ITB  At-risk populations   - Household contacts - Health workers - Migrants - People living with HIV - Children <5 years |
| External Framing | General perceptions  Media  Stigma  Gender |
| Severity | Severity in comparison to other issues |
| Credible indicators | Indicators   - Of prevalence - Of treatment success - Of adherence - Underreporting - Global burden of TB   Biomarkers |
| Effective interventions | Target Product Profile  Research gaps   - Basic science - Epidemiology - Qualitative/social science research - Implementation research   Suggested interventions   - Integrated models of care - Community engagement - Media campaign - Education campaign - Continuous medical education   Treatment   - Adherence - Equitable access - Proposed regimens |
| Guiding institutions | Guiding institutions  Private sector   - Innovation - Investment - Role - Engagement - Patents |
| Leadership | World Health Organization (WHO) |
| Civil society mobilization | Non-Governmental Organizations  Public-private partnerships  Patient Advocacy Groups |
| Global governance structure | Governance |
| Policy community cohesion | Policy community cohesion  Policy gaps |
| Policy windows | Policy window  Policy shift  Political environment |
| **Other** | |
| Predicted challenges | Risk communication  Accurate biomarkers |
| Ideal characteristics of a future ITB test and its implementation | Ideal setting  Ideal target groups  Cost-effectiveness  Health system integration  Ideal treatment regimens |
